# Characterizing Protease Specificity: How Many Substrates Do We Need?

**DOI:** 10.1371/journal.pone.0142658

**Published:** 2015-11-11

**Authors:** Michael Schauperl, Julian E. Fuchs, Birgit J. Waldner, Roland G. Huber, Christian Kramer, Klaus R. Liedl

**Affiliations:** Institute of General, Inorganic and Theoretical Chemistry, and Center for Molecular Biosciences Innsbruck (CMBI), University of Innsbruck, Innrain 80–82, A-6020 Innsbruck, Tyrol, Austria; Stanford University, UNITED STATES

## Abstract

Calculation of cleavage entropies allows to quantify, map and compare protease substrate specificity by an information entropy based approach. The metric intrinsically depends on the number of experimentally determined substrates (data points). Thus a statistical analysis of its numerical stability is crucial to estimate the systematic error made by estimating specificity based on a limited number of substrates. In this contribution, we show the mathematical basis for estimating the uncertainty in cleavage entropies. Sets of cleavage entropies are calculated using experimental cleavage data and modeled extreme cases. By analyzing the underlying mathematics and applying statistical tools, a linear dependence of the metric in respect to 1/n was found. This allows us to extrapolate the values to an infinite number of samples and to estimate the errors. Analyzing the errors, a minimum number of 30 substrates was found to be necessary to characterize substrate specificity, in terms of amino acid variability, for a protease (S4-S4’) with an uncertainty of 5 percent. Therefore, we encourage experimental researchers in the protease field to record specificity profiles of novel proteases aiming to identify at least 30 peptide substrates of maximum sequence diversity. We expect a full characterization of protease specificity helpful to rationalize biological functions of proteases and to assist rational drug design.

## Introduction

Proteases are enzymes that proteolytically cleave peptide bonds and account for around two percent of all human gene products [1]. Additionally, they account for one to five percent of the genome of infectious organisms, rendering them attractive drug targets [2]. Proteases are involved in a variety of physiological processes including food digestion [3] as well as complex signaling cascades such as for example the apoptosis pathway [4], the blood coagulation cascade [5] or the complement system [6].

The broad range of biological functions is reflected in highly specialized substrate specificities of proteases. While some proteases are highly promiscuous and cleave a variety of substrates, others show high specificity for particular substrate sequences [7]. Substrate specificity of a protease is determined by molecular interactions at the protein-protein interface of protease and substrate in the binding cleft of the protease. Amino acid side chains of the substrate are accommodated within subpockets of the protease. A unique nomenclature for the subpockets of proteases has been developed by Schechter and Berger [8]: The substrate's scissile bond is assigned between the residues P1 (N-terminal) and P1' (C-terminal), indices are incremented for further residues in both direction. Protease subpockets are numbered accordingly Sn-Sn', ensuring consistent indexing between interacting regions. Binding modes of substrate peptides are highly similar as the substrate is locked in an extended beta conformation in the binding cleft [9]. This arrangement typically involves residues P3-P3', in case of elastase even the P5 residue is tightly bound to the protease [10].

Several techniques have been developed to experimentally probe substrate specificity of proteases as reviewed by Poreba and Drag [11] as well as Diamond [12]. They include diverse experimental approaches based on chromatography [13], phage display [14], combinatorial substrate libraries [15, 16] as well as usage of fluorogenic substrates [17] and labeling techniques [18, 19]. The MEROPS database [20] hosts an annotated collection of protease cleavage sites of diverse experimental sources facilitating data mining and comparison of protease specificity [21]. Similar services with smaller data sets on proteolytic cleavage events are available via CutDB and PMAP [22, 23].

Recently, we have developed metrics to quantify, map, and compare protease specificity. Subpocket-wise cleavage entropies allow to quantify specificity of protease subpockets as well as overall specificity [24]. Cleavage entropies S_i_ are based on experimental substrate sequences from the MEROPS database. They are calculated as a Shannon entropy [25] over the probability of occurrence normalized to the natural occurrence p_a,i_ of amino acids a at each substrate position i. Cleavage entropies close to the maximum of one resemble unspecific substrate cleavage, whereas low values close to zero indicate stringent substrate recognition.

$$S_{i}=-\sum_{a=1}^{20}p_{a,i}{\text{log}}_{20}(p_{a,i})$$ (1)

Cleavage entropies were found helpful for direct comparison of substrate specificities of proteases, detection of sub-site cooperativities as well as tracing protease specificity along evolution [24]. Nevertheless it should be mentioned that the cleavage entropy is only measuring the promiscuity of the protease. To compare how similar the substrates of two protease are other metrics, like substrate similarity should be used [26]. We use the term substrate specificity as a measurement of substrate variability and not of substrate similarity. Furthermore it should be added that the cleavage entropy is measuring the promiscuity and not the sequence logo of a protease [27]. Molecular origins of protease specificity can be investigated based on subpocket-wise cleavage entropies, as they can directly be mapped to protease pockets and compared to local binding site characteristics [28]. Furthermore, substrate-guided techniques can be used to intuitively group proteases based on their binding preferences [26].

As all methods described rely on experimental substrate data, a critical assessment of the data basis is crucial. In the literature, the convergence behavior of entropy measurements has been published already decades ago [29, 30] and has been intensively studied since then up to now [31]. Different methods to correct the error due to finite samples, based on the statistics of information entropy, were reported [32–35]. These approaches are commonly used in a variety of fields not only including biologically and chemically relevant information like DNA sequences [36, 37] and neural spike trains [38], but also other data like the English language [39]. A common approach is to estimate the underlying probability function and use the result to estimate the entropy of the real probability function using rank-ordered histogram-based approaches [32, 40] or *Bayesian* statistics like approaches [41]. As estimating the probability distribution from a given sample can be complicated and computationally demanding, an easier and faster access to an infinite sample approximation is of general interest. In this work, a simple approach to correct the bias of the cleavage entropy due to a limited number of peptide samples is presented. The underlying mathematics are analyzed in order to come up with a mathematically valid approach, converging to the exact value for an infinite number of substrates. To further validate the model, test cases are analyzed, and the minimum number of substrates to characterize a protease in terms of subpocket-wise cleavage entropy is calculated. The performance is further compared with known entropy estimators from literature [33, 42]. To the best of our knowledge this is the first time that correction algorithms for finite samples are used in the context of protease substrate data.

## Methods

If the total cleavage behavior of a protease with eight subpockets (e.g. S4-S4'), including all natural possible octapeptides substrates (20 natural amino acids at each position), should be investigated, a total number of 20^8^ (= 25,600,000,000) substrates would have to be tested. Since this is practically not possible, the probabilities p of finding a specific AA at a specific position i in a substrate have to be estimated by testing a subset of these octapeptides and calculating estimated probabilities q. The empirical probability for an event k_a,i_ in our case the occurrence of the amino acid a in one of the eight pockets i, can be calculated as the quotient of occurrence of amino acid a, with the occurrence of any amino acid in this pocket (Eq 2) [43].

$$p(k_{a,i})\approx q(k_{a,i})=\frac{k_{a,i}}{\sum k_{a,i}}=\frac{k_{a,i}}{n}$$ (2)

The entropy measurement introduced above uses real probabilities p, but in practice only estimated probabilities q can be used. This simplification leads to two possible types of errors in the entropy measurement: Firstly, the statistical error of the metric, which can be expressed/measured by the variance [42]. Secondly, also a bias due to the limited number of samples is possible. So in the general case of any Shannon entropy based metric Eq 3 is true. The expectation value of the entropy cannot be split in the expectation value of the probability and the logarithmic probability.

$$\langle S_{i}\rangle \;=-\sum_{a=1}^{20}\langle q_{a,i}{\text{log}}_{20}q_{a,i}\rangle \;\neq -\sum_{a=1}^{20}\;\langle q_{a,i}\rangle \;\langle {\text{log}}_{20}q_{a,i}\rangle \;$$ (3)

The unequal sign would only become an equal sign if the values of q_a,i_ and log(q_a,i_) were independent from each other. This is not the case as the logarithmic function log(q_a,i_) is strictly monotonically increasing with q_a,i_ (positive correlation) resulting in a general underestimation of the entropy. The aim of this paper is to develop a method to reduce this systematic underestimation and also add a significance value to the estimated and already published values [24].

### Binomial distribution to analyze the underlying mathematics

To analyze the substrate variability of proteases, a mathematical description of the process is necessary. A way to mathematically describe the process of testing sampled substrates out of a larger set is the binomial distribution. In this ansatz, the experimental bias of the experimentalist, who most probably tends to test peptides similar to known substrates, or of the experiment itself, e.g. the predigesting process in proteomics [44], is neglected. The probability q_a,i_(k) of measuring k substrates with an amino acid a on the position i (e.g. P1) is a function of the total number of known substrates n and the real probability that this substrate is accepted in this pocket p_a,i_ (Eq 4). For all modelled data the natural occurrence of amino acids is neglected, but for the analysis of real proteases the probabilities are corrected for their abundance in the proteome [45].

$$q_{a,i}(k)=\left(\begin{array}{c} n\\ k \end{array}\right)p_{a,i}^{k}{\left(1-p_{a,i}\right)}^{n-k}$$ (4)

Inserting the probability function into the definition of the cleavage entropy (Eq 1) expansion and reordering of the terms leads to Eq 5.

$$\begin{array}{ll} \langle S_{i,n}\rangle & =-\overset{20}{\underset{a=1}{\sum }}q_{a,i}{\text{log}}_{20}\left(q_{a,i}\right)\\ & =-\overset{20}{\underset{a=1}{\sum }}p_{a,i}{\text{log}}_{20}\left(p_{a,i}\right)-\frac{1}{n}\overset{20}{\underset{a=1}{\sum }}\overset{n}{\underset{k=1}{\sum }}k{\text{log}}_{20}\left(\frac{k}{np_{a,i}}\right)\left(\begin{array}{c} n\\ k \end{array}\right)p_{a,i}^{k}{\left(1-p_{a,i}\right)}^{n-k} \end{array}$$ (5)

This equation provides a mathematical description for the expectation value of the measured entropy S_i,n_ as a function of the real entropy and an error term. S_i,n_ is defined as entropy calculated with the empirical probabilities without any correction algorithm, including n samples (the classically reported value). This term is further called the measured or naïve entropy. A detailed explanation how to derive Eq 5 is given in the Supporting Information.

The first term on the right hand side of the second equal sign corresponds to the "real entropy" or the entropy calculated with an infinite number of samples. In the following this term is called the real entropy or the infinite sample entropy. The second term on the right side of the equal sign describes the difference between the real entropy and the measured entropy, which corresponds to the error introduced due to limited sample size.

This term is further called the error or correction term. Moving the correction term in Eq 5 leads to an equation for the infinite sample entropy as a function of the measured entropy and the error term (Eq 6).

$$\begin{array}{ll} \langle S_{i,\infty }\rangle & =-\overset{20}{\underset{a=1}{\sum }}p_{a,i}{\text{log}}_{20}\left(p_{a,i}\right)\\ & =-\overset{20}{\underset{a=1}{\sum }}q_{a,i}{\text{log}}_{20}\left(q_{a,i}\right)+\frac{1}{n}\overset{20}{\underset{a=1}{\sum }}\overset{n}{\underset{k=1}{\sum }}k{\text{log}}_{20}\left(\frac{k}{np_{a,i}}\right)\left(\begin{array}{c} n\\ k \end{array}\right)p_{a,i}^{k}{\left(1-p_{a,i}\right)}^{n-k} \end{array}$$ (6)

### Using a linear regression to calculate the real entropy

The naïve entropy S_n_ can be calculated directly from the substrate data, as described by Fuchs et al. [24]. The still unknown term is the error term, which is investigated closer in the next paragraph.

**Fig 1:**
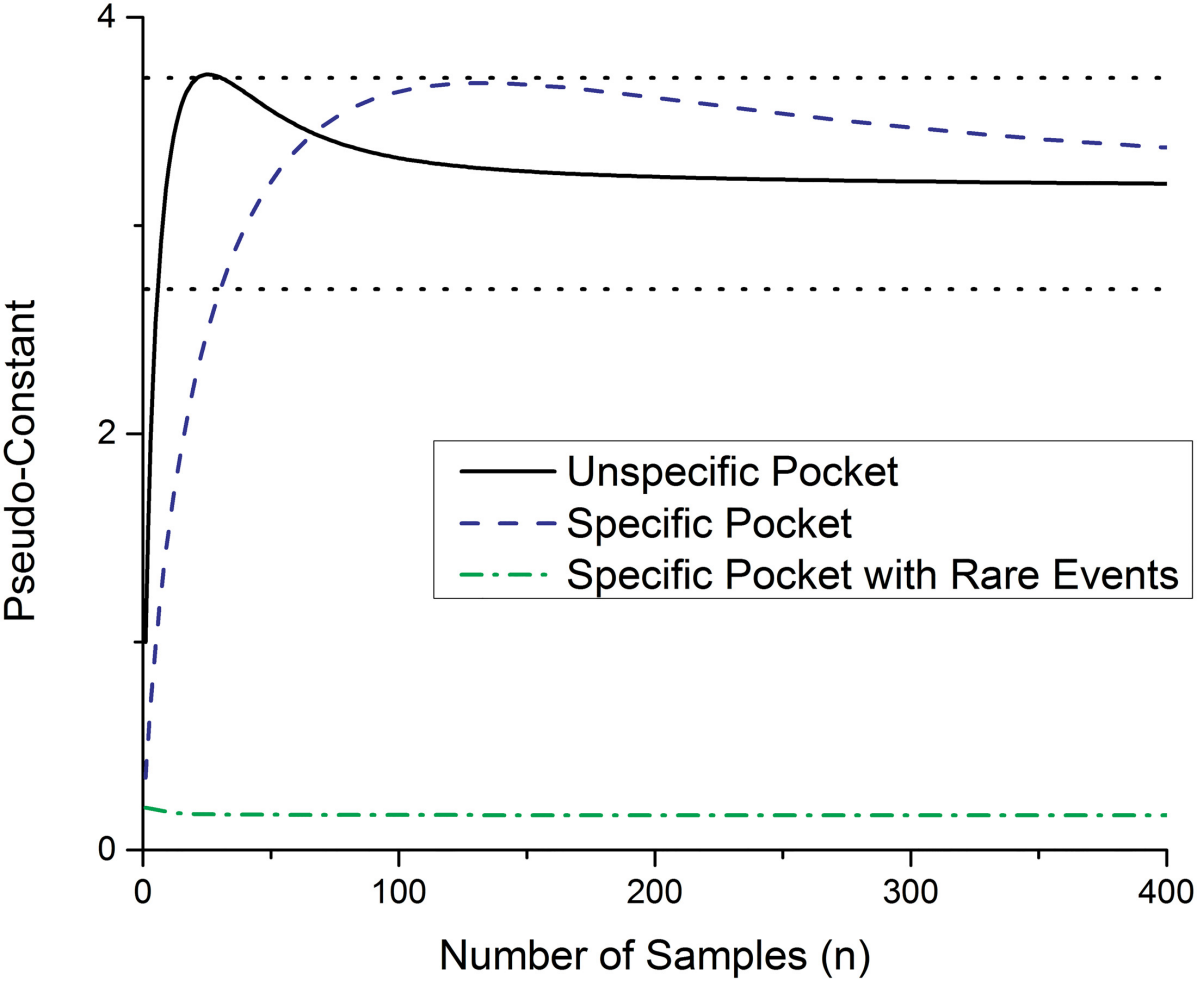
Dependence of the Pseudo-constant C with the number of samples n. The black full line indicates the value for an unspecific pocket, the black dotted lines indicate the region where the value of the Pseudo-constant is less than 15% off compared to the infinite number value. The green dashed dotted line shows the behavior of the constant for a specific pocket and the blue dashed line of a specific pocket with rare events (p<1%).

It is possible to split the error term into two parts. The first part only contains the scaling term 1/n and the second part the double-sum, which is further called “Pseudo-constant”. With a second order Taylor approximation of the logarithmic function it can be shown that the sum tends to be constant for a high value of samples n (for 100 samples with an equal distribution the error is smaller than four percent) and so the error term is a linear function with respect to the reciprocal number of samples [46]. To gain a better insight in the behavior of the term without looking in detail at the mathematics, the sum is plotted as a function of the number of samples in Fig 1.

The dependence of the Pseudo-constant on the probabilities p_a,i,_ and on the number of samples n, and the convergence with an increasing number of samples is presented in Fig 1. The convergence is slower for pockets with a very low probability to accept individual amino acids (18 AAs with 1% probability and 2 AAs with 41% probability; Fig 1: Specific pocket with rare events). Due to the low probability of these events, the influence on the calculated entropy is low. In the further manuscript we will prove that the most challenging case for the entropy measurement, in terms of convergence is the case of the unspecific pocket, where every amino acid has the same likelihood to appear in the pocket.

The linear behavior of the pseudo-constant can be used for a linear regression approach to remove the error term in Eq 5. This can be done by extrapolating the calculated entropy to 1/n equals 0, or in other words to the infinite sample value representing complete sampling of the substrate space.

The problem of the model is that the value of the Pseudo-constant is not known. A way around this problem is using a linear regression (Eq 7). One point of the regression is the naïve entropy S_n1_ calculated with all substrates n_1_. To create the second necessary point for the linear regression, bootstrapping is used [47], which means a random subset of substrates of size n_2_ is chosen and the entropy value S_n2_ for this subset is calculated. By repeating this process 100 times and using the average value a good approximation for a second data point can be created.

$$S_{\infty }=S_{n_{1}}\left(\frac{n_{1}}{n_{1}-n_{2}}\right)+S_{n_{2}}*\left(\frac{n_{2}}{n_{2}-n_{1}}\right)$$ (7)

The linear regression allows estimation of the real entropy value as the intercept of the measured entropy (in 1/n space). The dashed lines in Fig 1 are bordering the region where the value of the constant is less than 15% off compared to the value for an infinite number of samples in case of an unspecific pocket. This means when the constant is in that region, at least 85% of the systematic error is removed. To achieve this the minimum number of samples is 30.

### Error estimation—Variance analysis

The previous chapter shows that a large part of the systematic error can be removed by the approach presented. Nevertheless, it should be mentioned that only the systematic error is corrected by this approach. To predict a confidence interval of the entropy, the variance has to be taken into account. In general, a higher number of samples also reduces the uncertainty due to statistical fluctuations (variance).

The formula for the variance of a binomial distribution is known and by applying "Gauß's error propagation rules" the variance for the measured entropy can be calculated (formula 8) [46].

$$Var(S_{i}(n))=\sum_{a=1}^{20}\frac{q_{a,i}*(1-q_{a,i})}{n}{\left({\text{log}}_{20}q_{a,i}+S_{i}(n)\right)}^{2}$$ (8)

To calculate the uncertainty of the estimated value, again the "Gauß's error propagation rules" are applied to Eq 7. As the uncertainty of the data point created by bootstrapping cannot be smaller than the error of the data point using all samples, we assume that the standard deviation is the same for both points. The bootstrapping process is repeated 100 times, therefore the statistical error of this process is not significant compared to the error due to limited sampling.

$$\Delta S_{\infty }=\sqrt{{\left(\Delta S_{n_{1}}\frac{n_{1}}{n_{2}-n_{1}}\right)}^{2}+{\left(\Delta S_{n_{2}}*\frac{n_{2}}{n_{2}-n_{1}}\right)}^{2}}$$ (9)

By applying the presented rules for removing the systematic error of the entropy and by coming up with a definition for the variance it is possible to calculate corrected entropy values with a confidence interval. In other words, it is possible to predict how many substrates we need to significantly characterize a protease in terms of substrate specificity.

## Results

### Modeled extreme cases

Extreme cases of cleavage entropies were investigated with the program Mathematica [48]. Starting from a given probability function p, we analyzed the possible measured probabilities q and the values we got from applying the equations derived in the previous sections. Three different extreme cases were investigated, including the totally specific pocket, a specific pocket with rare events (pockets with p_a,i_ = 1%) and the totally unspecific pocket.

#### Totally specific pocket

In the extreme case of a totally specific pocket, only one amino acid (AA) is accepted, which means that the correct value is already known after one substrate is tested since measuring of negative events (substrates which cannot be cleaved) is not possible. The entropy of the pocket is zero and is not changing with an increasing number of samples. Also the uncertainty is always zero for this case. The presented method is also valid in this case (with a Pseudo-constant of zero for the linear fit).

**Fig 2:**
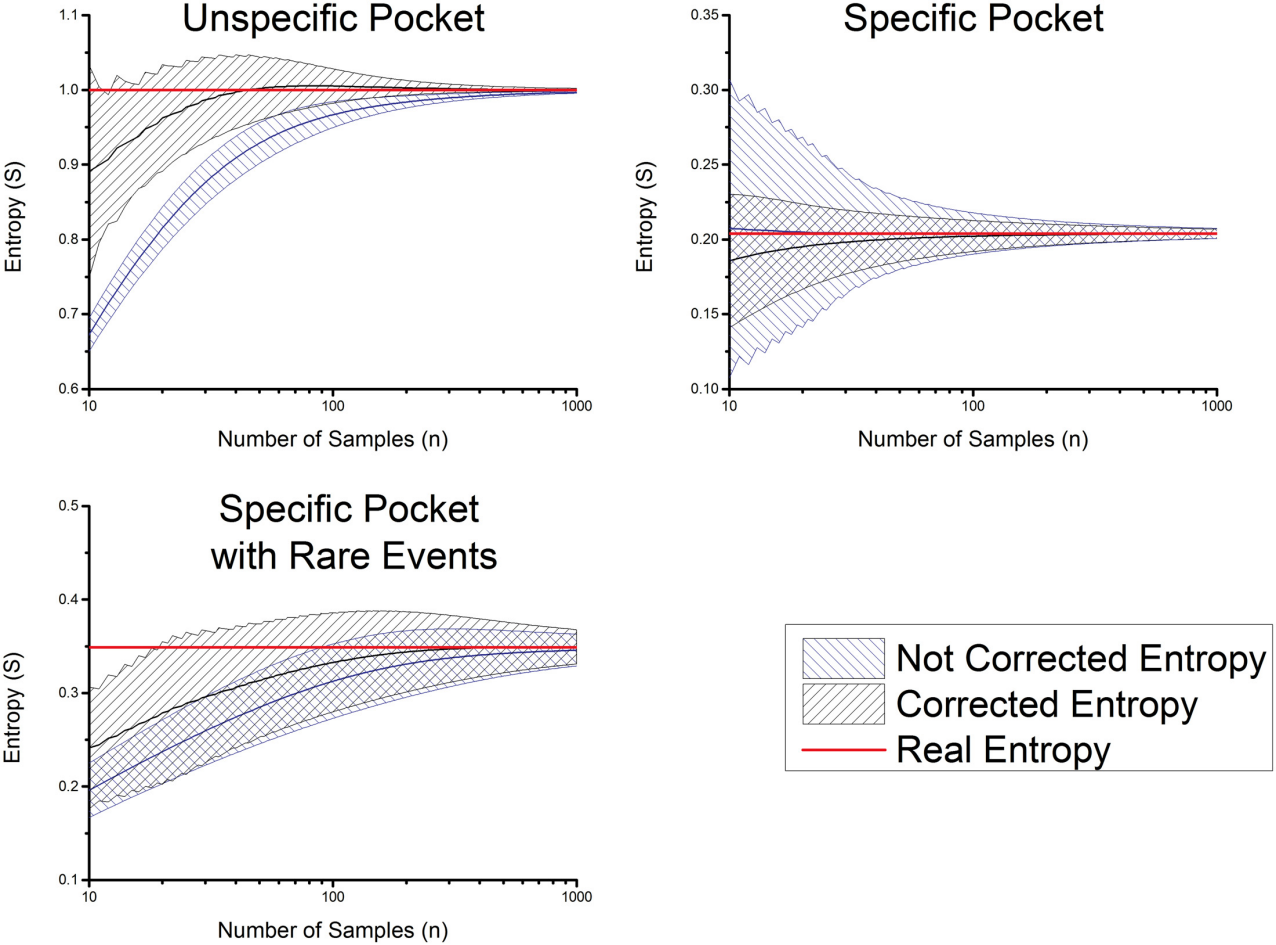
Trend of the measured entropy and estimated entropy with the number of known substrates. The cases of a totally unspecific pocket (upper left), an unspecific pocket (upper right) and an unspecific pocket with rare events (lower left) are shown. The filled areas correspond to the possible measured values including the standard deviation.

**Fig 3:**
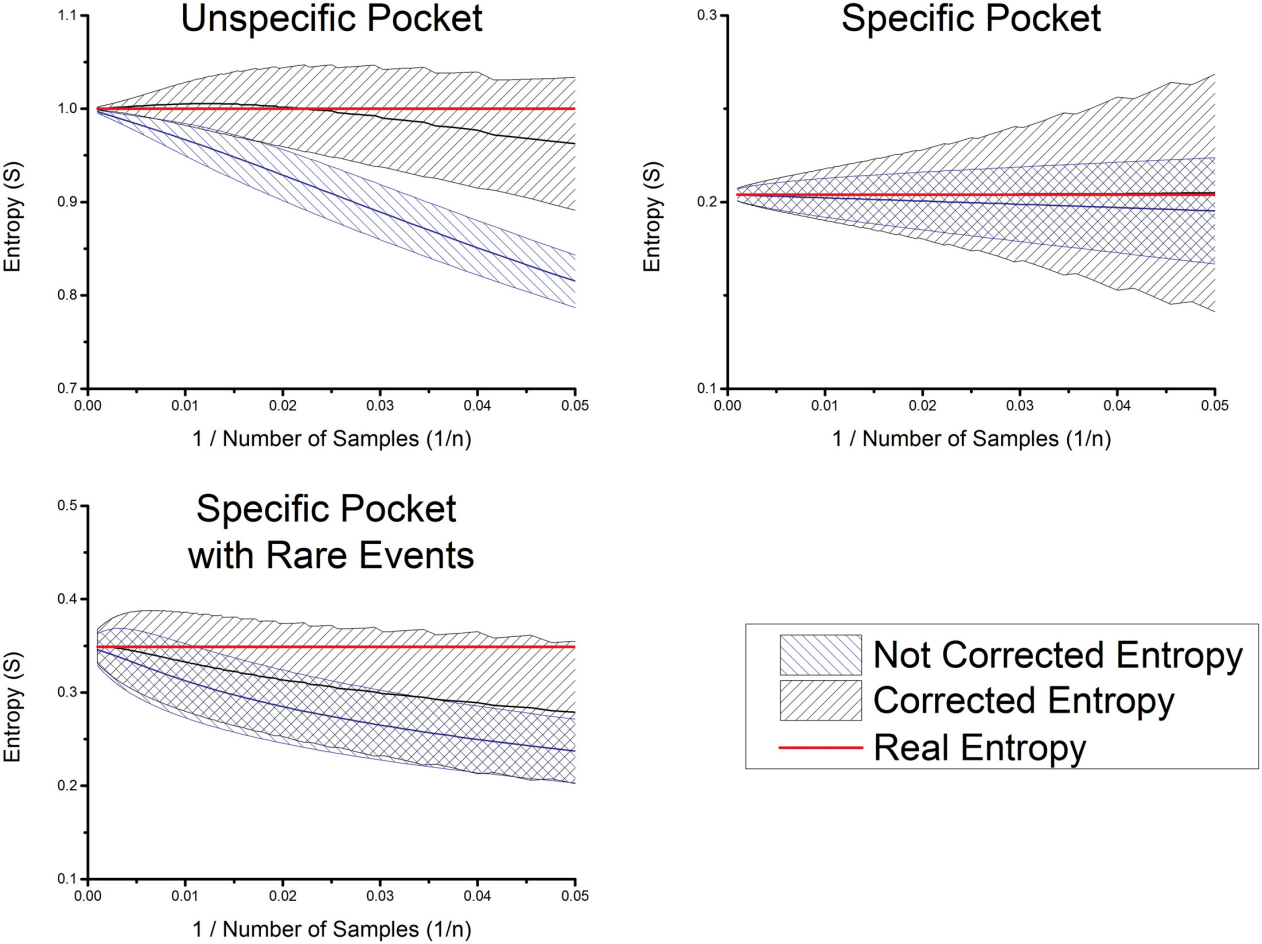
Trend of the measured and estimated entropy with the reciprocal number of known substrates. The cases of a totally unspecific pocket (upper left), an unspecific pocket (upper right) and an unspecific pocket with rare events (lower left) are shown. The filled areas correspond to the possible measured values including the standard deviation.

A probably more realistic scenario is a pocket with 70% probability for one AA and a 30% probability to find a second (different) AA in this pocket. In Fig 2 (upper right) the full line indicates the expectation values of the measured entropy; the shades are the confidence intervals (including one standard deviation) of the entropy plotted against the number of samples and the reciprocal value of the number of samples (Fig 3 upper right). The real space plot shows that the value is in close proximity to the real value already for a small number of samples. As expected, the reciprocal plot shows an almost linear behavior. This result shows that in the case of a very specific pockets it is not of major interest to improve the measured entropy values because the measured value and the real values are very close together. Already with a very low number of samples, e.g. number of substrates equals 20 the naïve entropy gives a reasonable result. Still, it should be noted that for only 253 out of 3999 proteases in MEROPS (9.12) more than 20 substrates are annotated.

#### Totally unspecific pocket

The most challenging case is the totally unspecific pocket. The totally unspecific pocket is a pocket in which every AA is found with the same probability, resulting in p_a,i_ = 0.05 for every AA. In comparison to the values for the specific pocket the error made for an unspecific pocket is significantly higher. This is the case where an extrapolation of the entropy value appears necessary. The correction algorithm presented in this paper shows a significant improvement compared to uncorrected values, as the majority of the systematic error is removed. It should be mentioned that the standard deviation increases; in particular for substrate numbers lower than 50. However, this is compensated by the improvement in the estimation of the expectation value. Furthermore, we show in the Supporting Information that the simplifications made by calculating the error lead to an overestimation of the mathematical expected standard deviation compared to the measured (statistical) standard deviation.

The reciprocal plot of the entropy (Fig 3 upper left) shows that a nearly linear dependence, in the reciprocal space of the substrates count, is given for more than 20 samples for the entropy of an unspecific pocket. This plot indicates that the value of the slope of the correction and in a further step the entropy will be underestimated in the region between 10 to 20 samples and between 20 to 50 samples slightly overestimated.

#### Rare events

The possibility of rare events results in a general underestimation of the correction factor. This is due to the slightly non-linear behavior of the estimated entropy (see Fig 1). Nevertheless, the results still improve compared to the uncorrected entropies (Fig 3 lower right).

The entropy value for an infinite number of substrates will also depend on how many samples n_2_ are used to create the subset for the second data point of the linear regression by the bootstrapping process. Two different factors have to be taken into account: If the number n_2_ is chosen very close to n_1_ or n (total number of samples), only a small Δx (Δn) value is used to calculate the slope, which means that also a small error in the value for Δy (ΔS) has a massive influence on the results. In contrast, if a value too far away is chosen, resulting in a small n_2_, the linear dependence is not given for the whole area. A closer look at this effect is presented in the Supporting Information. To summarize the results given there: For a small number of substrates a small ratio between n_2_ to n_1_ is favorable and for more samples in the database a higher ratio improves the result. Reasonable results can be achieved for all numbers of substrates by using the empirically derived formula 10.

$$n_{2}=\;\{\begin{array}{cc} \sqrt{5\cdot n_{1}} & x\geq 20\\ \frac{n1}{2} & x≺20 \end{array}$$ (10)

### Test case trypsin

**Fig 4:**
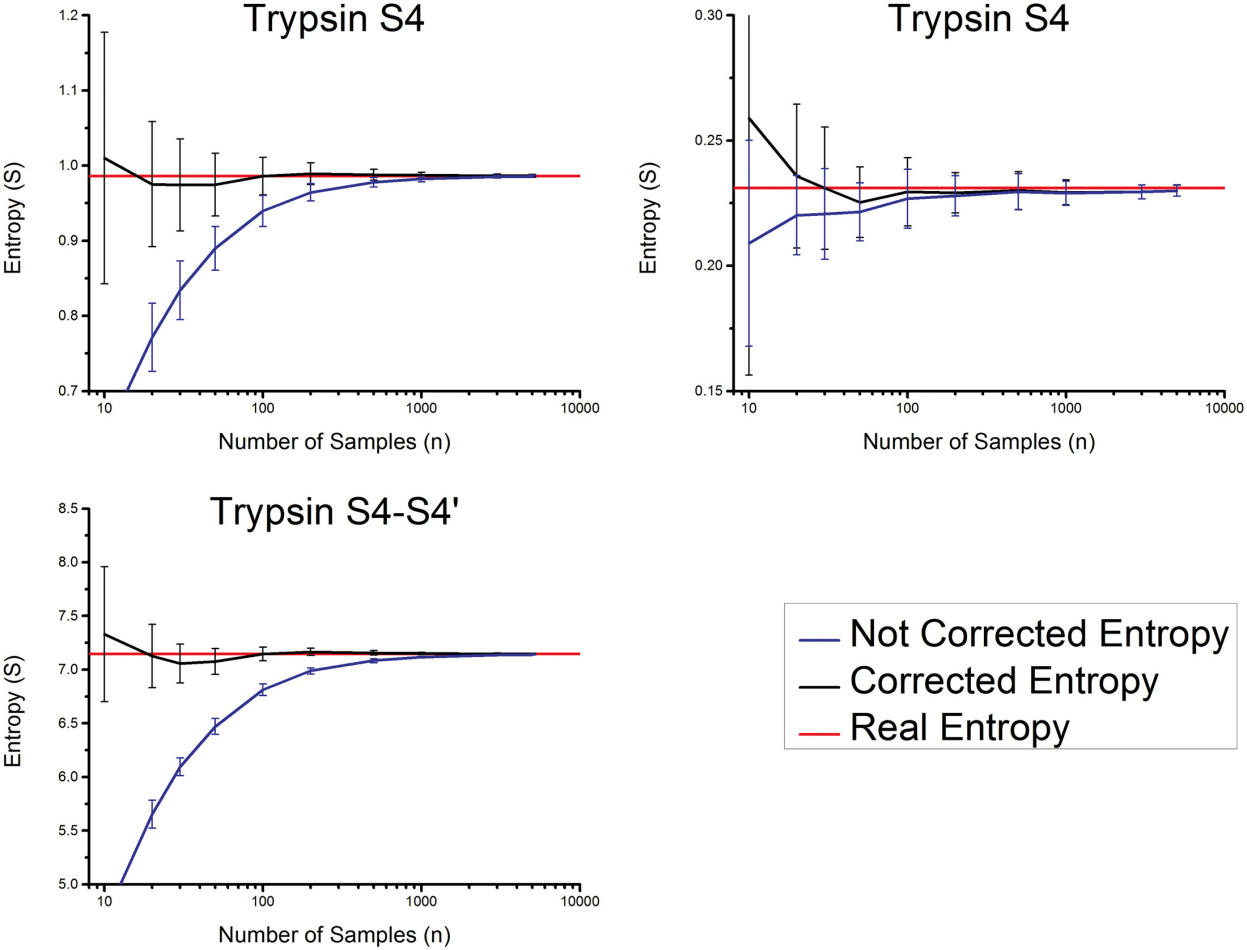
Trend of the naïve and estimated entropy for trypsin pocket S4, S1 and the sum of S4-S4’pockets. The behavior of the corrected entropy (black line) with the number of known substrates. The red line is the real/infinite sample entropy and the blue line corresponds to the naïve estimated entropy value. Trend is plotted for S4 (upper left), S1 (upper right), and the sum of S4 to S4’ (lower left).

The protease with the most entries in the MEROPS database is Trypsin-1. More than 10,000 known substrates are included in this database for the pockets S4 to S4’. It can be assumed that the values of the cleavage entropy for 10,000 substrates are very close to the values for an infinite number of substrates. The present approach is tested by taking a random subset of trypsin substrates and predicting the values based on these subsets. This procedure is repeated 1,000 times, allowing us to calculate a standard deviation and an average value. Subsets of 10, 20, 30, 50, 100, 200, 300, 500, 1000, 2000, 5000 and 10000 are taken from the data set. Comparison between the cleavage entropy with all known substrates and the expectation values from the subsets is shown in Fig 4. The statistically measured variance (plotted in Fig 4) is compared in the Supporting Information with the mathematically calculated variance.

Fig 4 demonstrates a slightly higher variance for the extrapolated values, but their mean value is significantly closer to the real value, especially for the case of a small substrate set for the description of the S1 (upper left). Almost the entire systematic error is gone compared to the measured value. To achieve a result which is only 10% off (confidence interval of 10%), 30 substrates (for the worst case of an unspecific pocket) are needed.

This again supports the finding that 30 substrates are necessary and sufficient to characterize the cleavage entropy of a protease, in a way that the specificity of a protease with an error less than ten percent can be predicted. For the total cleavage entropy of the protease trypsin we came even closer to the real value and are only off by 5% or less for 30 experimentally found substrates.

### Comparison of Trypsin—Thrombin—Factor Xa

By applying the formulas on different proteases we hope to get a broader insight into the specificity of these proteases. For that case we are looking at the digestive enzyme trypsin and two enzymes involved in the blood coagulation cascade, factor Xa (fXa) and thrombin, 3 proteases with similar substrate preferences [49]. Without applying correction algorithms it is easy to see that trypsin has only one selective pocket S1. The sub-pocket-wise cleavage entropies for all other pockets are higher than 0.98, which means they are very close to be completely unspecific. To decide if the pockets for different proteases are significantly different, the corrected values are compared. For thrombin we also have a specific S1 pocket but also the pockets S2 and the S1' site show specificity, whereas all other pockets show nearly no specificity. Comparing the two blood coagulation proteins and their subpocket-wise variabilities (see Table 1), a significant difference in variability is found for the pockets S3 and S2’, whereas the other 5 pockets show no statistically significant difference. These two pockets are more selective in fXa compared to thrombin.

**Table 1:** Naïve and extrapolated cleavage entropies for Trypsin, Thrombin and Factor Xa: The cleavage entropies for these three proteases are given for the 8 subpockets S4-S4’ and the total (sum) cleavage entropy. Data from MEROPS (9.12).

Protein	Known Substates (n)	Entropy	S4	S3	S2	S1	S1'	S2'	S3'	S4'	Total
**Trypsin**	**14083**	**not corrected**	**0.986**	**0.991**	**0.99**	**0.231**	**0.975**	**0.993**	**0.991**	**0.99**	**7.146**
			±0.001	±0.001	±0.001	±0.001	±0.001	±0.001	±0.001	±0.001	±0.002
		**corrected**	**0.986**	**0.991**	**0.99**	**0.231**	**0.975**	**0.993**	**0.991**	**0.99**	**7.149**
			±0.001	±0.001	±0.001	±0.001	±0.001	±0.001	±0.001	±0.001	±0.003
**Thrombin**	**185**	**not corrected**	**0.892**	**0.971**	**0.635**	**0.176**	**0.754**	**0.937**	**0.901**	**0.945**	**6.211**
			±0.014	±0.01	±0.025	±0.022	±0.019	±0.009	±0.013	±0.011	±0.046
		**corrected**	**0.917**	**0.998**	**0.650**	**0.178**	**0.783**	**0.957**	**0.930**	**0.974**	**6.387**
			±0.018	±0.012	±0.030	±0.026	±0.025	±0.013	±0.016	±0.014	±0.057
**fXa**	**59**	**not corrected**	**0.787**	**0.788**	**0.57**	**0.132**	**0.72**	**0.731**	**0.859**	**0.779**	**5.368**
			±0.034	±0.031	±0.034	±0.025	±0.032	±0.039	±0.025	±0.028	±0.089
		**corrected**	**0.851**	**0.835**	**0.607**	**0.137**	**0.791**	**0.789**	**0.935**	**0.848**	**5.793**
			±0.049	±0.045	±0.048	±0.035	±0.056	±0.068	±0.044	±0.050	±0.142

### Comparison to other estimators

**Fig 5:**
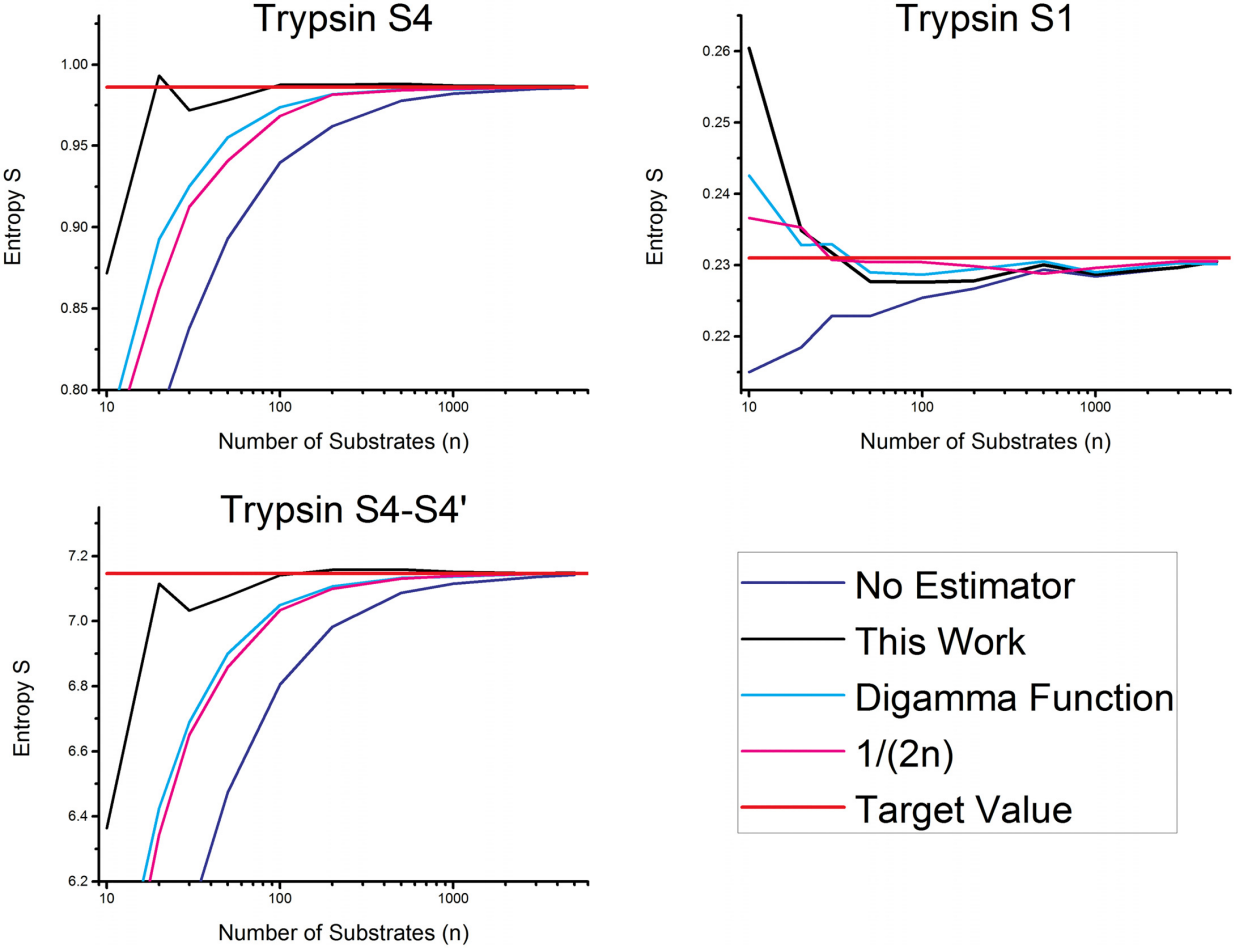
Comparison of the different entropy estimators. The estimation process presented in this work is outperforming the compared published estimators.

In this paragraph the estimator presented in this work is compared to known entropy estimators [31, 33, 35, 50, 51]. A detailed description of Bayesian entropy estimation approach, which seems to be not suitable for this problem is given in the Supporting Information. Therefore substrate subsets of trypsin are taken from the MEROPS database and the entropy is calculated with those different estimators. Fig 5 shows the result as a function of the substrate number (number of samples: logarithmic axis). Top left shows the behavior for the unspecific pocket S4. The worst assumption for this pocket is the uncorrected entropy just by applying the formula for substrate entropy without any correction estimator. A significant improvement is the addition of the square root of samples to correct the term. A slightly better result can be achieved by using the digamma function. A further modification of the estimator published by Grassberger and co-workers [33] gives again only a slightly better value. Our estimator is significantly outperforming the other estimators in a range between 30 to 100 samples. This is the most interesting range for estimating substrate entropies. If we have less than 30 samples, it is not possible to describe the behavior of the protease correctly and for more than 100 substrates the cleavage entropy is already well estimated and the difference between estimators is negligible. So the presented approach can reduce the amount of substrates needed for estimating the correct values. Once more we want to highlight the fact that unselective pockets are the hardest to describe accurately in terms of cleavage entropy.

In contrast to the S4, the pocket S1 is a specific pocket only allowing the accommodation of two different substrate amino acids. For this pocket all estimators are performing equally well. Our estimator shows the biggest deviation for a sample number of 10. As a substrate number of 10 substrates is too low to characterize substrate specificity, this value can be neglected. The overall cleavage entropy, over all eight pockets, is well estimated by our estimator. The rank order and the values are very similar to the case of the S4 pocket. As seven out of the eight pockets are similar to S4 this result is the logic consequence. Analysis of degradation enzymes like trypsin are the hardest cases, as these enzymes are the most unspecific. Enzymes involved in signal processes with a higher specificity can be described with the same number of substrates at least equally well.

## Discussion

Initially, proteases were simply seen as protein-degrading enzymes, showing only limited substrate selectivity. More recently, their importance for cellular signaling processes has been reported [52]. It is important to understand proteases in terms of specificity and variability more accurately in order to understand the possible interactions in the signaling pathways. Furthermore, a protease cannot be seen independently, instead proteolytic enzymes have to be seen in a more global context as they influence each other in cascades, also determined by specificity [53].

These properties render proteases attractive targets for drug discovery. One of the main problems with protease drug targets is to selectively hit one single protease [54]. In order to achieve this, a better understanding of substrate specificity is crucial. In this paper the convergence behavior of the cleavage entropy as a metric for protease specificity is investigated and based on the results a new estimation process for a limited number of samples is tested. The substrate independent estimator of this work makes it possible to compare proteases in terms of specificity with different amounts of experimentally found substrates. This easier comparison can facilitate drug design processes as specificity and selectivity are key aspects in drug design, reducing side effects.

**Fig 6:**
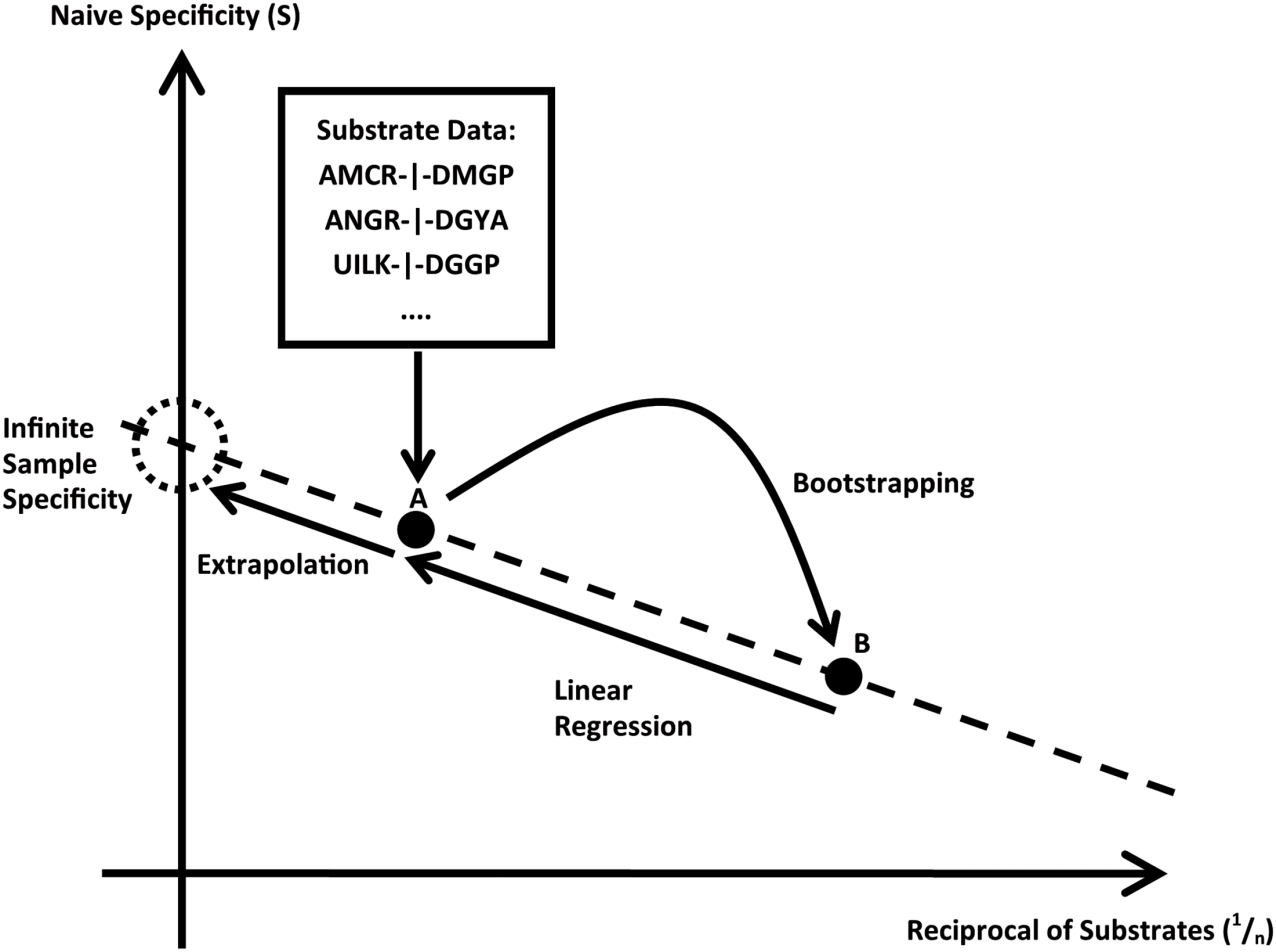
Systematic sketch of the estimation process for the corrected cleavage entropy. Based on experimental substrate data the specificity A is calculated. Through bootstrapping a subset is created and the specificity of this subset is calculated to generate B. By performing a linear fit and extrapolating the specificity to 0 in 1/n space we estimate the specificity for infinite substrates.

Fig 6 schematically shows the process of entropy correction presented in this contribution. Using the set of substrate sequences as input, the data point A is calculated by applying the equation for the naïve substrate entropy (formula 1). Using bootstrapping, a second data set was created and by applying the same formula on this subset the point B was created. Due to the linear convergence behavior of the entropy with the reciprocal substrate number ($\frac{1}{n}$), a linear regression based on these two points may be performed. By extrapolating the regression towards ($\frac{1}{n}\;=\;\emptyset )\;$ it is possible to extract the estimated infinite sample substrate entropy as the intercept of the vertical axis. A Microsoft Excel Macro is provided, allowing the reader to enter substrate data and obtain the corrected entropies based on the algorithm presented in this work.

The new estimator outperforms the tested estimators from the literature. Based on the analysis and the convergence behavior of the estimator and the corresponding variance we found that a minimum of 30 substrates is required to reliable calculate the exact cleavage entropy value with a maximum error of 10%. Therefore, we encourage experimental researchers in the protease field to record specificity profiles of novel proteases aiming to identify at least 30 peptide substrates of maximum sequence diversity.

Similar problems of entropy estimation from finite sample size occur not only in the field of protease research. The problem of finite substrate samples occurs in all disciplines of the ‘omics field, including systems like single nucleotide polymorphism [55], endonucleases [56], ribonucleases [57] and transcription factors [58]. The presented extrapolation technique can be expanded to most of these cases and the critical review of the convergence behavior should be able to support statistical analysis in these fields.

## Supporting Information

S1 Fig Comparison of the statistical calculated standard deviation and the mathematical derived standard deviation. Mathematical standard deviation was calculated according to Eq 1 using the average value of 100 subsamples. The entropy variances for the naïve estimation (left) and for entropies employing our correction algorithm (right) are presented.
(TIF)

S2 Fig Comparison of the different bootstrapping subsets for the trypsin test case. Different subsets sizes for bootstrapping were tested. For low substrate numbers a smaller ratio between total substrate number and subset substrates lead to better results. However, for higher total substrate values the opposite is the case. The corrected entropy values using different subset sizes are shown for the substrate position S4 (upper-left), S1 (upper right), and the sum of S4-S4’ (bottom left).
(TIF)

S3 Fig Comparison of the different priors for the Bayesian statistics approach. Different values of β (prior weight) were tested. A high value of β is hindering the metric to get correct values for specific pockets, but a too low value of β is not improving the results significantly. The obtained entropy values calculated with different β values are shown for the substrate position S4 (upper-left), S1 (upper right), and the sum of S4-S4’ (bottom left).
(TIF)

S1 File ProteaseSpecificityCalculator. Excel sheet including macros to calculate naïve and corrected cleavage entropies.
(XLSM)

S1 Text Error comparison, derivation of Eq 5, influence of bootstrapping subset size, and Bayesian entropy estimation. (PDF)

## References

[1] Puente XS, Sanchez LM, Gutierrez-Fernandez A, Velasco G, Lopez-Otin C. A genomic view of the complexity of mammalian proteolytic systems. Biochem Soc Trans. 2005;33(Pt 2):331–4. 15787599 10.1042/BST0330331

[2] Madala PK, Tyndall JD, Nall T, Fairlie DP. Update 1 of: Proteases universally recognize beta strands in their active sites. Chem Rev. 2010;110(6):PR1–31. 10.1021/cr900368a 20377171

[3] Richter C, Tanaka T, Yada RY. Mechanism of activation of the gastric aspartic proteinases: pepsinogen, progastricsin and prochymosin. Biochem J. 1998;335 (Pt 3):481–90. 9794784 10.1042/bj3350481 PMC1219805

[4] Hengartner MO. The biochemistry of apoptosis. Nature. 2000;407(6805):770–6. 11048727 10.1038/35037710

[5] Davie EW, Fujikawa K, Kisiel W. The coagulation cascade: initiation, maintenance, and regulation. Biochemistry. 1991;30(43):10363–70. 1931959 10.1021/bi00107a001

[6] Muller-Eberhard HJ. Molecular organization and function of the complement system. Annu Rev Biochem. 1988;57:321–47. 3052276 10.1146/annurev.bi.57.070188.001541

[7] Hedstrom L. Introduction: Proteases. Chem Rev. 2002;102(12):4429–30. 10.1021/cr000033x 12475199

[8] Schechter I, Berger A. On the size of the active site in proteases. I. Papain. Biochem Biophys Res Commun. 1967;27(2):157–62. 6035483 10.1016/s0006-291x(67)80055-x

[9] Tyndall JD, Nall T, Fairlie DP. Proteases universally recognize beta strands in their active sites. Chem Rev. 2005;105(3):973–99. 15755082 10.1021/cr040669e

[10] Hedstrom L. Serine Protease Mechanism and Specificity. Chem Rev. 2002;102(12):4501–24. 12475199 10.1021/cr000033x

[11] Poreba M, Drag M. Current strategies for probing substrate specificity of proteases. Curr Med Chem. 2010;17(33):3968–95. 20939826 10.2174/092986710793205381

[12] Diamond SL. Methods for mapping protease specificity. Curr Opin Chem Biol. 2007;11(1):46–51. 17157549 10.1016/j.cbpa.2006.11.021

[13] O'Donoghue AJ, Eroy-Reveles AA, Knudsen GM, Ingram J, Zhou M, Statnekov JB, et al Global identification of peptidase specificity by multiplex substrate profiling. Nat Methods. 2012;9(11):1095–100. 10.1038/nmeth.2182 23023596 PMC3707110

[14] Matthews DJ, Wells JA. Substrate phage: selection of protease substrates by monovalent phage display. Science. 1993;260(5111):1113–7. 8493554 10.1126/science.8493554

[15] Turk BE, Huang LL, Piro ET, Cantley LC. Determination of protease cleavage site motifs using mixture-based oriented peptide libraries. Nat Biotechnol. 2001;19(7):661–7. 11433279 10.1038/90273

[16] Boulware KT, Daugherty PS. Protease specificity determination by using cellular libraries of peptide substrates (CLiPS). Proc Natl Acad Sci U S A. 2006;103(20):7583–8. 16672368 10.1073/pnas.0511108103 PMC1456804

[17] Harris JL, Backes BJ, Leonetti F, Mahrus S, Ellman JA, Craik CS. Rapid and general profiling of protease specificity by using combinatorial fluorogenic substrate libraries. Proc Natl Acad Sci U S A. 2000;97(14):7754–9. 10869434 10.1073/pnas.140132697 PMC16617

[18] Mahrus S, Trinidad JC, Barkan DT, Sali A, Burlingame AL, Wells JA. Global sequencing of proteolytic cleavage sites in apoptosis by specific labeling of protein N termini. Cell. 2008;134(5):866–76. 10.1016/j.cell.2008.08.012 18722006 PMC2566540

[19] Schilling O, Overall CM. Proteome-derived, database-searchable peptide libraries for identifying protease cleavage sites. Nat Biotechnol. 2008;26(6):685–94. 10.1038/nbt1408 18500335

[20] Rawlings ND, Waller M, Barrett AJ, Bateman A. MEROPS: the database of proteolytic enzymes, their substrates and inhibitors. Nucleic Acids Res. 2014;42(Database issue):D503–9. 10.1093/nar/gkt953 24157837 PMC3964991

[21] Rawlings ND. A large and accurate collection of peptidase cleavages in the MEROPS database. Database (Oxford). 2009;2009:bap015. 20157488 10.1093/database/bap015 PMC2790309

[22] Igarashi Y, Eroshkin A, Gramatikova S, Gramatikoff K, Zhang Y, Smith JW, et al CutDB: a proteolytic event database. Nucleic Acids Res. 2007;35(Database issue):D546–9. 17142225 10.1093/nar/gkl813 PMC1669773

[23] Igarashi Y, Heureux E, Doctor KS, Talwar P, Gramatikova S, Gramatikoff K, et al PMAP: databases for analyzing proteolytic events and pathways. Nucleic Acids Res. 2009;37(Database issue):D611–8. 10.1093/nar/gkn683 18842634 PMC2686432

[24] Fuchs JE, von Grafenstein S, Huber RG, Margreiter MA, Spitzer GM, Wallnoefer HG, et al Cleavage Entropy as Quantitative Measure of Protease Specificity. PLoS Comput Biol. 2013;9(4):e1003007 10.1371/journal.pcbi.1003007 23637583 PMC3630115

[25] Shannon CE. A Mathematical Theory of Communication. Bell System Technical Journal,. 1948;27:379–423, 623–56.

[26] Fuchs JE, von Grafenstein S, Huber RG, Kramer C, Liedl KR. Substrate-driven mapping of the degradome by comparison of sequence logos. PLoS Comput Biol. 2013;9(11):e1003353 10.1371/journal.pcbi.1003353 24244149 PMC3828135

[27] Schneider TD, Stephens RM. Sequence logos: a new way to display consensus sequences. Nucleic Acids Res. 1990;18(20):6097–100. 2172928 10.1093/nar/18.20.6097 PMC332411

[28] Fuchs JE, von Grafenstein S, Huber RG, Wallnoefer HG, Liedl KR. Specificity of a protein-protein interface: local dynamics direct substrate recognition of effector caspases. Proteins. 2014;82(4):546–55. 10.1002/prot.24417 24085488 PMC4282588

[29] Miller GA. Note on the bias of information estimates. 1955 p. 95–100.

[30] Tarasenko FP. On the evaluation of an unknown probability density function, the direct estimation of the entropy from independent observations of a continuous random variable, and the distribution-free entropy test of goodness-of-fit. Proc IEEE. 1968;56(11):2052–3.

[31] Györfi L, van der Meulen EC. Density-free convergence properties of various estimators of entropy. Comput Stat Data Anal. 1987;5(4):425–36.

[32] Beirlant J, Dudewicz EJ, Györfi L, Meulen EC. Nonparametric Entropy Estimation: An Overview. International Journal of the Mathematical Statistics Sciences. 1997;6:17–39.

[33] Grassberger P. Entropy Estimates from Insufficient Samplings. ARXIV. 2003.

[34] Schmitt AO, Herzel H, Ebeling W. A new method to calculate higher-order entropies from finite samples. Europhys Lett. 1993;23(5):303–9.

[35] Schurmann T, Grassberger P. Entropy estimation of symbol sequences. Chaos. 1996;6(3):414–27. 12780271 10.1063/1.166191

[36] Herzel H, Grosse I. Measuring correlations in symbol sequences. Physica A. 1995;216(4):518–42.

[37] Schmitt AO, Herzel H. Estimating the entropy of DNA sequences. J Theor Biol. 1997;188(3):369–77. 9344742 10.1006/jtbi.1997.0493

[38] Nemenman I, Bialek W, van Steveninck RD. Entropy and information in neural spike trains: Progress on the sampling problem. Phys Rev E. 2004;69(5):6. 10.1103/PhysRevE.69.056111 15244887

[39] Wagner AB, Viswanath P, Kulkarni SR. Probability Estimation in the Rare-Events Regime. IEEE Trans Inf Theory. 2011;57(6):3207–29.

[40] Pöschel T, Ebeling W, Frömmel C, Ramírez R. Correction algorithm for finite sample statistics. Eur Phys J E. 2003;12(4):531–41. 15007750 10.1140/epje/e2004-00025-4

[41] Holste D, Grosse I, Herzel H. Bayes' estimators of generalized entropies. J Phys A. 1998;31(11):2551–66.

[42] Bonachela JA, Hinrichsen H, Munoz MA. Entropy estimates of small data sets. J Phys A. 2008;41(20):9.

[43] Kolmogorov AN. On Logical Foundations of Probability Theory In: Prokhorov J, Itô K, editors. Probability Theory and Mathematical Statistics. Lecture Notes in Mathematics. 1021: Springer Berlin Heidelberg; 1983 p. 1–5.

[44] Meissner F, Mann M. Quantitative shotgun proteomics: considerations for a high-quality workflow in immunology. Nat Immunol. 2014;15(2):112–7. 10.1038/ni.2781 24448568

[45] McCaldon P, Argos P. Oligopeptide biases in protein sequences and their use in predicting protein coding regions in nucleotide sequences. Proteins. 1988;4(2):99–122. 3227018 10.1002/prot.340040204

[46] Roulston MS. Estimating the errors on measured entropy and mutual information. Physica D. 1999;125(3–4):285–94.

[47] Efron B. Bootstrap Methods: Another Look at the Jackknife. 1979:1–26.

[48] Wolfram Research I. Mathematica Version 10.1. Wolfram Research, Inc 2015;Champaign, Illinois.

[49] Nar H, Bauer M, Schmid A, Stassen JM, Wienen W, Priepke HW, et al Structural basis for inhibition promiscuity of dual specific thrombin and factor Xa blood coagulation inhibitors. Structure. 2001;9(1):29–37. 11342132 10.1016/s0969-2126(00)00551-7

[50] Grassberger P. Finite-sample corrections to entropy and dimension estimates. Phys Lett A. 1988;128(6–7):369–73.

[51] Ilya Nemenman FS, William Bialek. Entropy and inference, revisited. Advances in Neural Information Processing Systems. 2002;14:NECI TR 2001–067, NSF-ITP-02-02.

[52] Turk B. Targeting proteases: successes, failures and future prospects. Nat Rev Drug Discov. 2006;5(9):785–99. 16955069 10.1038/nrd2092

[53] Fortelny N, Cox JH, Kappelhoff R, Starr AE, Lange PF, Pavlidis P, et al Network Analyses Reveal Pervasive Functional Regulation Between Proteases in the Human Protease Web. PLoS Biol. 2014;12(5):e1001869 10.1371/journal.pbio.1001869 24865846 PMC4035269

[54] Drag M, Salvesen GS. Emerging principles in protease-based drug discovery. Nat Rev Drug Discov. 2010;9(9):690–701. 10.1038/nrd3053 20811381 PMC2974563

[55] Ji H, Liu XS. Analyzing 'omics data using hierarchical models. Nat Biotech. 2010;28(4):337–40. 10.1038/nbt.1619 PMC2904972 20379180

[56] Pattanayak V, Lin S, Guilinger JP, Ma E, Doudna JA, Liu DR. High-throughput profiling of off-target DNA cleavage reveals RNA-programmed Cas9 nuclease specificity. Nat Biotech. 2013;31(9):839–43. 10.1038/nbt.2673 PMC3782611 23934178

[57] Griffin MA, Davis JH, Strobel SA. Bacterial Toxin RelE: A Highly Efficient Ribonuclease with Exquisite Substrate Specificity Using Atypical Catalytic Residues. Biochemistry. 2013;52(48):8633–42. 10.1021/bi401325c 24251350 PMC3910103

[58] Kielbasa S, Gonze D, Herzel H. Measuring similarities between transcription factor binding sites. BMC Bioinformatics. 2005;6(1):237. 16191190 10.1186/1471-2105-6-237 PMC1261160

