# Supplementary figures and images for: Characterizing Protease Specificity: How Many Substrates Do We Need?
Source: PLoS One. 2015 Nov 11;10(11):e0142658. doi: 10.1371/journal.pone.0142658 (PMC4641643; doi:10.1371/journal.pone.0142658)

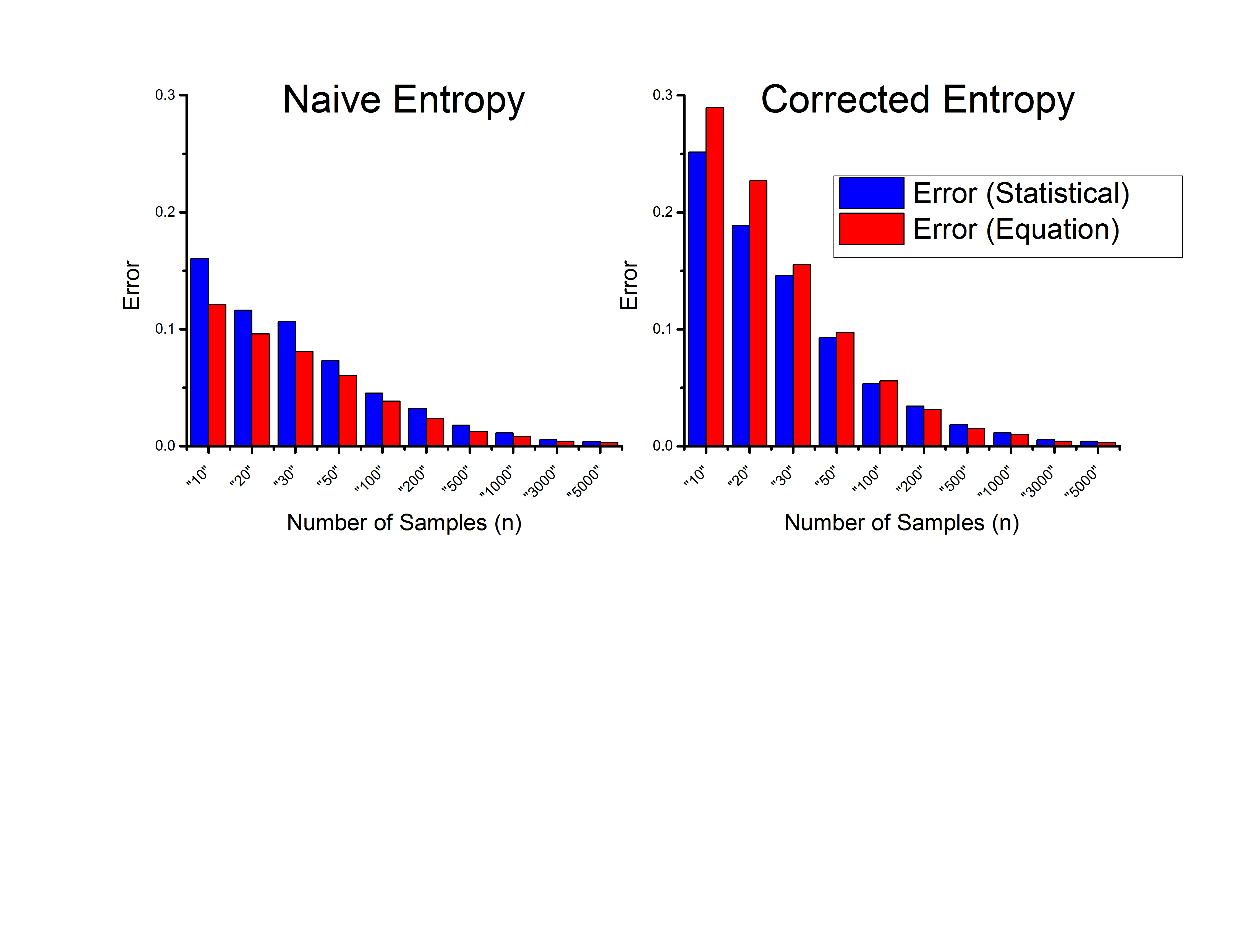

Supplement: S1 Fig — Mathematical standard deviation was calculated according to Eq 1 using the average value of 100 subsamples. The entropy variances for the naïve estimation (left) and for entropies employing our correction algorithm (right) are presented. (TIF) [file pone.0142658.s001.tif]

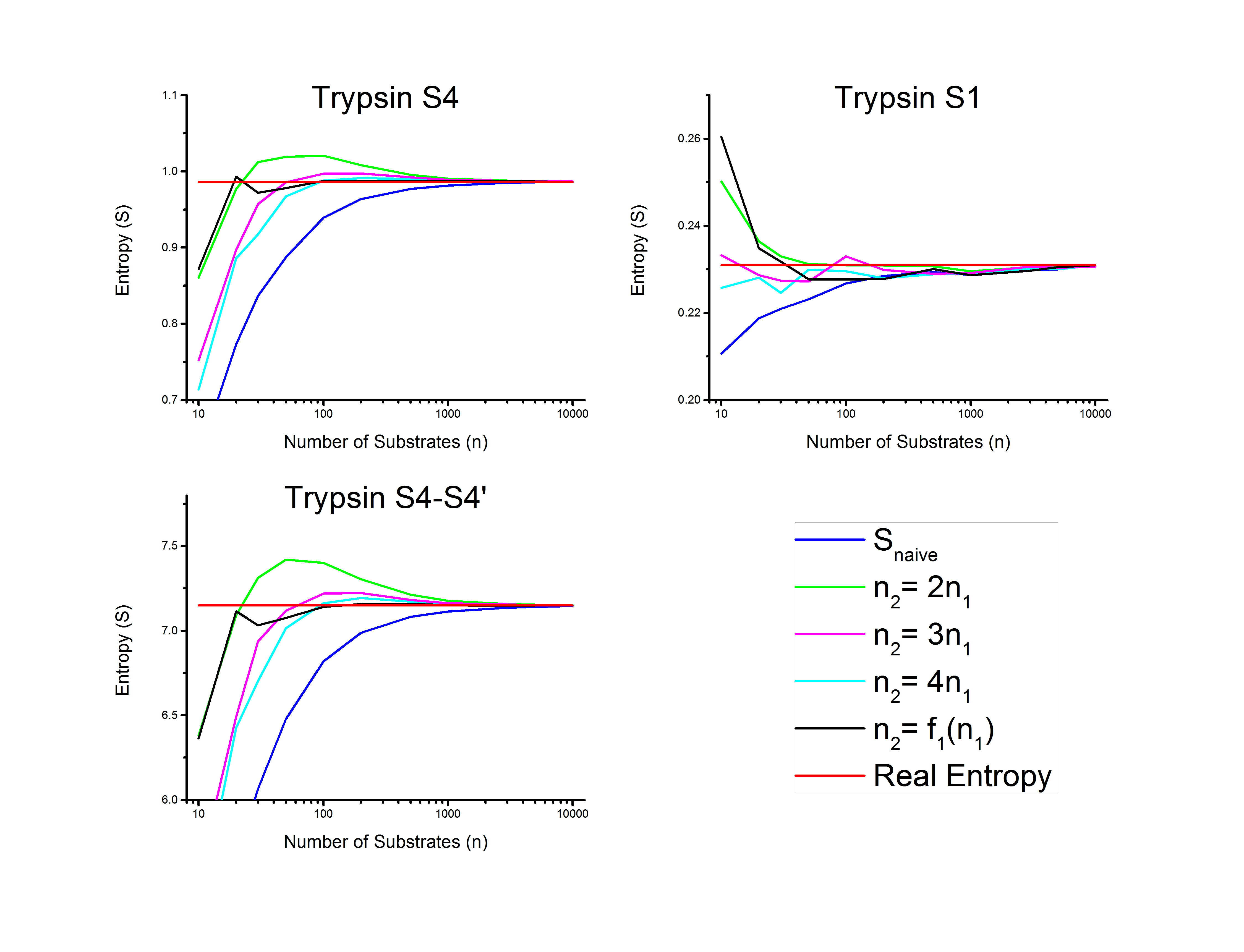

Supplement: S2 Fig — Different subsets sizes for bootstrapping were tested. For low substrate numbers a smaller ratio between total substrate number and subset substrates lead to better results. However, for higher total substrate values the opposite is the case. The corrected entropy values using different subset sizes are shown for the substrate position S4 (upper-left), S1 (upper right), and the sum of S4-S4’ (bottom left). (TIF) [file pone.0142658.s002.tif]
