# Supplementary material for: Characterizing Protease Specificity: How Many Substrates Do We Need?
Source: PLoS One. 2015 Nov 11;10(11):e0142658. doi: 10.1371/journal.pone.0142658 (PMC4641643; doi:10.1371/journal.pone.0142658)
Supplement: S1 Text — (PDF) [file pone.0142658.s005.pdf]

Supporting Information

Characterizing Protease Specificity: How many Substrates Do We Need?

Michael Schauperl[1], Julian E. Fuchs[1]*, Birgit J. Waldner[1], Roland G. Huber[1,2], Christian Kramer[1,3] and Klaus R. Liedl[1]

[1] Institute of General, Inorganic and Theoretical Chemistry, and Center for Molecular Biosciences Innsbruck (CMBI), University of Innsbruck, Innrain 80-82, A-6020 Innsbruck, Tyrol, Austria

[2] Present Address: Bioinformatics Institute (BII), Agency for Science, Technology and Research (A*STAR), #07-01 Matrix, 30 Biopolis Street, 138671 Singapore

[3] Present Address: Pharma Research and Early Development, Therapeutic Modalities, Roche Innovation Center Basel, Grenzacherstrasse 74, 4070 Basel, Switzerland.

*E-Mail corresponding author: Julian.Fuchs@uibk.ac.at

Comparison of calculated and derived error values

Like in the main article, it is assumed that the cleavage entropy of trypsin calculated from the complete substrate data set is the correct value. In this part the statistical error (variance) of the calculated entropies for trypsin is investigated. As shown by Roulsten [1], the entropy variance is expressed by the following equation:

$$Var(S_i(n)) = \sum_{a=1}^{20} \frac{p_{a,i} * (1 - p_{a,i})}{n} (\log_{20} q_{a,i} + S_i(n))^2 \quad (1)$$

n is the number of substrates taken to calculate the entropy, $q_{a,i}$ the probability for the amino acid a in pocket i, and $S_i(n)$ the calculated entropy value from the subset for pocket i. The variance calculated by equation 1 (mathematical approach) is compared to the standard deviation obtained through repeated subsampling of the full data set (statistical approach). The respective results are compared in Fig S1 for the uncorrected (left) and the corrected values (right).

Fig S1: Comparison of the statistical calculated standard deviation and the mathematically derived standard deviation. Mathematical standard deviation was calculated according to equation 1 using the average error of 100 independent subsamples. The entropy variances for the naïve estimation (left) and for entropies employing our correction algorithm (right) are presented.

Values calculated with either method are in good agreement with each other, thus demonstrating that standard deviations calculated by equation 1 are reasonably accurate. Applying the mathematical equation slightly underestimates the true variance of naive entropy. The opposite trend is observed for the error of the estimated values (Fig S1, right). Therefore we conclude that

the standard deviation of the uncorrected values is slightly higher than assumed in the main article for the modeled extreme cases and for the test case trypsin. Statistical error estimation for the corrected entropy is smaller than assumed for the modeled extreme cases and the test case trypsin. This result would mean that the error bars for the naïve entropy in Figs 4, 5, 6 in the main article are too small and the error bars for the estimated entropy too high. We therefore conclude that the differences between the variance of the naïve estimated entropy and the estimated entropy is overestimated in the main article.

Derivation of equation 5

The probability to find amino acid a in a pocket i is given by $p_{a,i}$, n defines the number of total samples and $k_{a,i}$ the instances where amino acid a is found in pocket i. With this mathematical probability function it is possible to estimate the errors and calculate the expectation value.

$$p(k_{a,i}) \approx q(k_{a,i}) = \frac{k_{a,i}}{\sum k_{a,i}} = \frac{k_{a,i}}{n} \tag{2}$$

Inserting the knowledge-based definition of the probability function (2) into the definition of the cleavage entropy S_i for pocket i (equation 1 main article), leads to an equation for the expectation value of cleavage entropy as a function of the total sample number n. $k_{a,i}$ is defined as the occurrence of amino acid a in pocket i.

$$E(S_i(n)) = E\left(\sum_{a=1}^{20} \frac{k_{a,i}}{n} \log_{20} \frac{k_{a,i}}{n} \right) \tag{3}$$

According to the linearity of the expectation value we can exchange the expectation value and the sums as following:

$$E\big(S_i(n)\big) = -\sum_{a=1}^{20} E\left(\frac{k_{a,i}}{n} \log_{20} \frac{k_{a,i}}{n} \right) \tag{4}$$

As outlined in the main article, we are using the binomial distribution (equation 5) to mathematically model the process of experimentally finding new substrates. The probability $q_{a,i}(k)$ of identifying k substrates having amino acid a in pocket i (e.g. S1) is a function of the total number of substrates found and the probability that this amino acid is accepted in this pocket.

$$q_{a,i}(k) = \binom{n}{k} p_{a,i}^{k} (1 - p_{a,i})^{n-k} \tag{5}$$

Insertion of the binomial distribution in equation 4 leads to equation 6.

$$E\left(S_i(n)\right) = -\frac{1}{n}\sum_{a=1}^{20}\sum_{k=1}^{n} k\log_{20}\left(\frac{k}{n}\right)\binom{n}{k} p_{a,i}^{k}(1-p_{a,i})^{n-k} \tag{6}$$

It is now possible to expand the fraction in the logarithmic function by n*$p_{a,i}$ and splitting up the terms according to log(a/b)=log(a)-log(b).

$$\begin{aligned} E\left(S_i(n)\right) = &-\frac{1}{n}\sum_{a=1}^{20}\log_{20}\left(p_{a,i}\right)\sum_{k=1}^{n} k\binom{n}{k} p_{a,i}^{k}(1-p_{a,i})^{n-k} \\ &+\sum_{k=1}^{n} k\log_{20}\left(\frac{k}{np_{a,i}}\right)\binom{n}{k} p_{a,i}^{k}(1-p_{a,i})^{n-k} \end{aligned} \tag{7}$$

In a further step we can move the log ($p_{a,i}$) in front of the sum over k, resulting in the expectation value of the binomial distribution (n*$p_{a,i}$) as the first term:

$$E\left(S_i(n)\right) = -\frac{1}{n}\sum_{a=1}^{20}\log_{20}\left(p_{a,i}\right) n * p_{a,i} - \frac{1}{n}\sum_{a=1}^{20}\sum_{k=1}^{n} k\log_{20}\left(\frac{k}{np_{a,i}}\right)\binom{n}{k} p_{a,i}^{k}(1-p_{a,i})^{n-k} \tag{8}$$

So the first term can be simplified to the definition of the cleavage entropy with the underlying probability distribution by reducing n in the first term, leading us to the final result (equation 5 in the paper):

$$\begin{aligned} E\left(S_i(n)\right) &= -\sum_{a=1}^{20}\log_{20}\left(q_{a,i}\right) q_{a,i} \\ &= -\sum_{a=1}^{20}\log_{20}\left(p_{a,i}\right) p_{a,i} - \frac{1}{n}\sum_{a=1}^{20}\sum_{k=1}^{n} k\log_{20}\left(\frac{k}{np_{a,i}}\right)\binom{n}{k} p_{a,i}^{k}(1-p_{a,i})^{n-k} \end{aligned} \tag{9}$$

Influence of the bootstrapping subset size on the entropy metric

n_2 is the number of samples used to create the second data point of our linear regression by bootstrapping. As our metric is dependent on the size of the subset, different ratios were tested and we finally came up with the empirically derived formula:

$$n_2 = \begin{cases} \sqrt{5 \cdot n_1} & x \geq 20 \\ \dfrac{n1}{2} & x \prec 20 \end{cases} \qquad (10)$$

Fig S2 shows the expectation value as a function of the number of substrates for different tested subset sizes. The function $f_1(n_1)$ was used in the main text. For a low substrate number a bigger subset for bootstrapping yields improved results. The larger subset is required as otherwise the linear approximation for the entropy metric is not valid. For a higher number of substrates a comparatively smaller subset improves the results, as it provides a higher Δn value and thus higher numerical stability of the regression.

Fig S2: Comparison of the different bootstrapping subsets for the trypsin test case. Different subsets sizes for bootstrapping were tested. For low substrate numbers a smaller ratio between total substrate number and subset substrates lead to better results. However, for higher total substrate values the opposite is the case. The corrected entropy values using different subset sizes are shown for the substrate position S4 (upper-left), S1 (upper right), and the sum of S4-S4' (bottom left).

Bayesian entropy estimation approach

The selection of the prior is crucial for the obtained results. We choose a Laplacian type prior as described by Nemenman [2], normalized to the natural occurrence of the amino acids. The non-normalised probabilities are calculated according to equation 11:

$$q_{a,i} = \frac{n_{a,i} + \beta * NO_a}{(N_i + \kappa) * NO_a}, \qquad \kappa = 20 * \beta \tag{11}$$

$n_{a,i}$ is the number of amio acids a in pocket i, N_i the total number of substrates known for pocket i, NO_a the relative natural occurrence of amino acid a. The parameter β can be interpreted as an extra amino acid occurrence/count (for β=1) or as a fraction of an extra count (β<1) in every bin/pocket. The results calculated with different values of β are presented in Fig S3

Fig S3: Comparison of the different priors for the Bayesian statistics approach. Different values of β (prior weight) were tested. A high value of β is hindering the metric to get correct values for specific pockets, but a too low value of β is not improving the results significantly. The obtained entropy values calculated with different β values are shown for the substrate position S4 (upper-left), S1 (upper right), and the sum of S4-S4' (bottom left).

References

1. Roulston MS. Estimating the errors on measured entropy and mutual information. Physica D. 1999;125(3–4):285-94.

2. Ilya Nemenman FS, William Bialek. Entropy and inference, revisited. Advances in Neural Information Processing Systems. 2002;14:NECI TR 2001-067, NSF-ITP-02-02.
